# Low Oxygen Tension Enhances Osteogenic Potential of Bone Marrow-Derived Mesenchymal Stem Cells with Osteonecrosis-Related Functional Impairment

**DOI:** 10.1155/2015/950312

**Published:** 2015-01-27

**Authors:** Lihong Fan, Ruiyu Liu, Jia Li, Zhibin Shi, Xiaoqian Dang, Kunzheng Wang

**Affiliations:** Department of Orthopedics, The Second Affiliated Hospital of Xi'an Jiaotong University, Xiwu Road, Xi'an, Shaanxi 710004, China

## Abstract

*Objective.* Glucocorticoids can affect the function of bone marrow-derived mesenchymal stem cells (BMMSCs) adversely and merit the requirement for a strategy to correct this anomaly; we assessed the effect of low oxygen (2%) on BMMSCs from rabbits with osteonecrosis. *Methods.* Bone marrow-derived mesenchymal stem cells from normal rabbits and rabbits with osteonecrosis were divided into four groups: (1) normal-normoxia group, with normal BMMSCs cultured under 20% oxygen; (2) osteonecrosis-normoxia group, with BMMSCs from rabbits with osteonecrosis cultured under 20% oxygen; (3) osteonecrosis-low oxygen treated group, with BMMSCs from rabbits with osteonecrosis cultured under 2% oxygen; (4) normal-low oxygen treated group, with normal BMMSCs cultured under 2% oxygen. The proliferation, osteogenic, and adipogenic differentiation of MSCs and expression of stemness genes, osteogenic, and adipogenic differentiation markers were investigated. *Results.* Compared with BMMSCs from normal rabbits, those from osteonecrosis rabbits showed significantly reduced proliferation ability, repressed expression of stemness genes, decreased osteoblasts formation, and increased adipocytes formation, indicating an osteonecrosis-related impairment. Low oxygen (2%) treated BMMSCs from osteonecrosis rabbits showed not only increased proliferation and osteogenic potential but also decreased adipogenic potential. *Conclusion.* Low oxygen (2%) culture represents a novel strategy to augment BMMSC function affected by glucocorticoids and holds significance for future strategies to treat femoral head osteonecrosis.

## 1. Introduction

Corticosteroid-induced osteonecrosis of the femoral head (ONFH) is one of the most serious complications induced by high dosages and/or long-term administration of steroid hormones. For early treatment, many surgical procedures are designed to preserve the femoral head; however, the results are unpredictable and the clinical outcomes are not favorable [1, 2].

Mesenchymal stem cells (MSCs) possess multilineage differentiation potential and have been widely used in the regeneration of damaged tissues including cardiovascular disorders, nerve injury, bone defect [3, 4], and osteonecrosis of the femoral head [5]. Even though MSCs represent a feasible choice for osteonecrosis repair, the effect of MSCs transplantation is still not fully satisfactory [6]. The possible explanation for the not good effect of MSCs transplantation is that the number as well as function of MSCs to repair damaged tissues declines with steroid administration and alcohol abuse. MSCs from osteonecrosis patients exhibit impaired survival, proliferation, and differentiation and therefore require a strategy to improve their repair function [7, 8].

A number of strategies have been used to enhance depleted stem cell function by treatment with growth factors, hypoxic shock, and antiaging compounds [9, 10]. Beneficial effects of low oxygen culture on proliferation and* in vitro* and* in vivo* differentiation potentials of MSCs have been suggested by several groups [11, 12]. However, the influences of low oxygen on differentiation of MSCs from osteonecrosis animals still remain not yet clear.

In the present study, effect of corticosteroid on bone marrow-derived mesenchymal stem cells (BMMSCs) was investigated and the effects of low oxygen (2%) on the proliferation ability and differentiation potentials of BMMSCs from osteonecrosis rabbits were examined.

## 2. Methods

### 2.1. Establishment of the Osteonecrosis Model

The male New Zealand white rabbits (age: 28 weeks; body weight: 2.5–3 kg) we used in the experiment were from the Experimental Animal Center of Xi'an Jiaotong University, China. The animals were housed under standard conditions at the Experimental Animal Center of Xi'an Jiaotong University and received a standard laboratory diet. The experimental protocols adhered to the NIH Guide for the Care and Use of Laboratory Animals and were approved by the Animal Ethical Committee of the Xi'an Jiaotong University. The rabbit model of early osteonecrosis of the femoral head (ONFH) was induced by methods according to the previously published protocols described by Qin et al. [13]. Briefly, one injection of 10 *μ*g/kg body weight of lipopolysaccharide (Sigma, St. Louis, MO, USA) was given intravenously. 24 hours later, three injections of 20 mg/kg body weight of methylprednisolone (Pfizer, USA) were given intramuscularly, at a time interval of 24 h. Osteonecrosis gradually developed 6 weeks after injection of MPS.

### 2.2. Bone Marrow Mesenchymal Stem Cell Cultures

Bone marrow-derived mesenchymal stem cells were harvested from normal rabbits and rabbits with ONFH. Briefly, rabbit marrow (1 mL) was collected and mononuclear cells were obtained by Ficoll-Paque density-gradient centrifugation (10.77 g/cm^3^). Cells of passages 3–5 were utilized for studies. To confirm the cellular identity of cultured cells, MSCs were subjected to fluorescence-activated cell sorting using CD90, CD34, and CD45 markers, and cultured cells were identified as CD90 positive and CD34/CD45 negative cells.

### 2.3. Low Oxygen Protocol and Normoxia Control

To establish a low oxygen model of culturing MSCs* in vitro*, MSCs were incubated in a special chamber, which could monitor the humidity, temperature, and the oxygen tension (pO_2_) automatically. According to different O_2_ concentration and different MSCs sources, cells were divided into four groups: (1) normal-normoxia group (N Group), with normal MSCs cultured under 20% oxygen; (2) osteonecrosis-normoxia group (O Group), with MSCs from rabbits with ONFH cultured under 20% oxygen; (3) osteonecrosis-low oxygen treated group (O-L Group), with MSCs from rabbits with ONFH cultured under 2% oxygen; (4) normal-low oxygen treated group (N-L Group), with normal MSCs cultured under 2% oxygen. Cells of each group were subjected to the same procedures except the different O_2_ concentration during the whole duration of study.

### 2.4. WST-1 Cell Proliferation Assay

In order to assess MSCs proliferation, cell proliferation was measured by a commercial WST-1 Kit (Beyotime Institute of Biotechnology, Shanghai Province, China). Briefly, 2 × 10^4^ cells were cultured in 96-well plates for 16 h, 10 *μ*L WST-1 was added to each well, and cells were cultured for additional 4 h in a humidified atmosphere. Then, the plate was shaked thoroughly for 1 min. The absorbance was determined as the proliferation rate using a 96-well plate reader at 450 nm (Sunrise Remote/Touch screen, Tecan, Austria). The optical density (OD) value of normal group was used as 100% cell proliferation. The experiment was independently performed three times.

### 2.5. Colony-Forming Unit (CFU) Assay

The MSCs were seeded on 10 cm tissue culture-treated polystyrene dishes at a density of 50 × 10^3^ cells/cm^2^. The culture medium was changed every 3 days. At day 7, cells were washed with PBS, fixed in 10% formalin, incubated with Giemsa dye for 10 min, and washed three times with PBS. The results are expressed as the mean number of CFU per 10^6^ of seeded MSCs, as well as the mean diameter of the CFU. In another 10 cm tissue culture-treated polystyrene dish, cultures were digested using 0.25% trypsin and the cells were counted using a hemocytometer.

### 2.6. Adipogenesis* In Vitro* Oil Red O Staining

MSCs were plated in 6-wells at a density of 10^5^ cells/cm^2^. For differentiation of MSCs to adipocytes, 10 mg/mL insulin, 0.2 mM indomethacin, 1 mM dexamethasone, and 0.5 mM 3-isobutyl-1-methyl-xanthine were used to supplement DMEM. The cells in normal-normoxia group and osteonecrosis-normoxia group were exposed to 20% oxygen under adipogenic differentiation conditions, and the cells in osteonecrosis-low oxygen treated group and normal-low oxygen treated group were exposed to 2% oxygen. After 21 days of adipogenic induction, Oil Red O staining was accomplished according to the procedures provided by the manufacturers of the Oil Red O staining kit. For quantitative analysis of adipogenesis, Oil Red O dye was eluted by 100% isopropanol and the absorbance of the resulting solution was measured at 540 nm by spectrophotometery.

### 2.7. Osteogenesis* In Vitro* ALP Staining and Alizarin Red S Staining

For osteogenic differentiation, MSCs were plated in 6-wells at a density of 2 × 10^4^ cells/cm^2^. In the case of differentiation in the osteogenic linage, DMEM was supplemented with 10 mM *β*-glycerophosphate, 10 nM dexamethasone, and 0.1 mM L-ascorbic acid-2-phosphate. The cells in normal-normoxia group and osteonecrosis-normoxia group were exposed to 20% oxygen under osteogenic differentiation conditions, and the cells in osteonecrosis-low oxygen treated group and normal-low oxygen treated group were exposed to 2% oxygen. Differentiation was terminated after 14 days and ALP staining was performed by using an alkaline phosphatase kit according to the manufacturer's instructions (Promega, Southampton, UK). The resulting blue, insoluble, granular dye deposit indicated sites of alkaline phosphatase activity. For Alizarin Red S staining, cells were fixed in 10% formalin for 30 minutes and stained with 2% Alizarin Red S solution for 30 minutes. Subsequently, cells were rinsed once with PBS at room temperature.

### 2.8. Immunofluorescence Staining

Two weeks after osteogenic induction, MSCs were fixed and treated with 4, 6-diamidino-2-phenyl-indol dihydrochloride (DAPI) 50 *μ*g/mL for nuclear staining. Then, these cells were stained for osteocalcin (OCN), type I collagen (COL I) antibody (Santa Cruz, CA) visualized with a TRITC-conjugated secondary antibody. The primary antibody was diluted 1 : 100; controls included stainings without primary antibody. Fluorescence images were obtained using a fluorescence microscope (Fluoview 400, Olympus).

### 2.9. Real-Time PCR

Total RNA of cells was prepared by using the Trizol reagent (Invitrogen, CA, USA) according to the manufacturer's specifications. After reverse transcription reaction, template DNA was then used in gene-specific PCR for Oct4, Nanog, PPAR*γ*-2, OCN, and COL I. The following primers were used: GAPDH, sense: 5′-CCACTTTGTGAAGCTCATTTCCT-3′, antisense: 5′-TCGTCCTCCTCTGGTGCTCT-3′; Oct4, sense: 5′-CGAGTGAGAGGCAACTTGG-3′, antisense: 5′-CGGTTACAGAACCACACACG-3′; Nanog, sense: 5′-CCCAGCTGTGTGTGCTCAA-3′, antisense: 5′-CCAGGCTTGGGAGTACCAGG-3′; PPAR-*γ*, sense: 5′-AGTCGCCATCCGCATCTT-3′, antisense: 5′-ATCTCATGGACGCCGTACTTG-3′; OCN, sense: 5′-TCACTCTTGTCGCCCTGCT-3′, antisense: 5′-CCTCCCTCTTGGACACGAA-3′; COL I, sense: 5′-TTGACAGAGGCGAACTGAGG-3′, antisense: 5′-AGAAAACCACACAACACAGAGGAG-3′. Gene-specific primers were synthesized commercially (Shengong Co. Ltd., Shanghai, China). Glyceraldehyde 3-phosphate dehydrogenase (GAPDH) served as a housekeeping gene. The conditions of real-time PCR were as follows: 40 cycles at 94°C for 5 s, 60°C for 34 s. Dissociation stage was added to the end of amplification procedure. There was no nonspecific amplification determined by the dissolve curve.

### 2.10. Statistical Analysis

All data are presented as mean ± standard deviation (SD). Statistical analysis was performed using Student's *t*-test. A *P* value less than or equal to 0.05 was considered significant for all statistical analyses.

## 3. Results

### 3.1. Low Oxygen Culture Promoted Colony Formation and Increased Proliferation of MSCs from ONFH Rabbits

**Figure 1:**
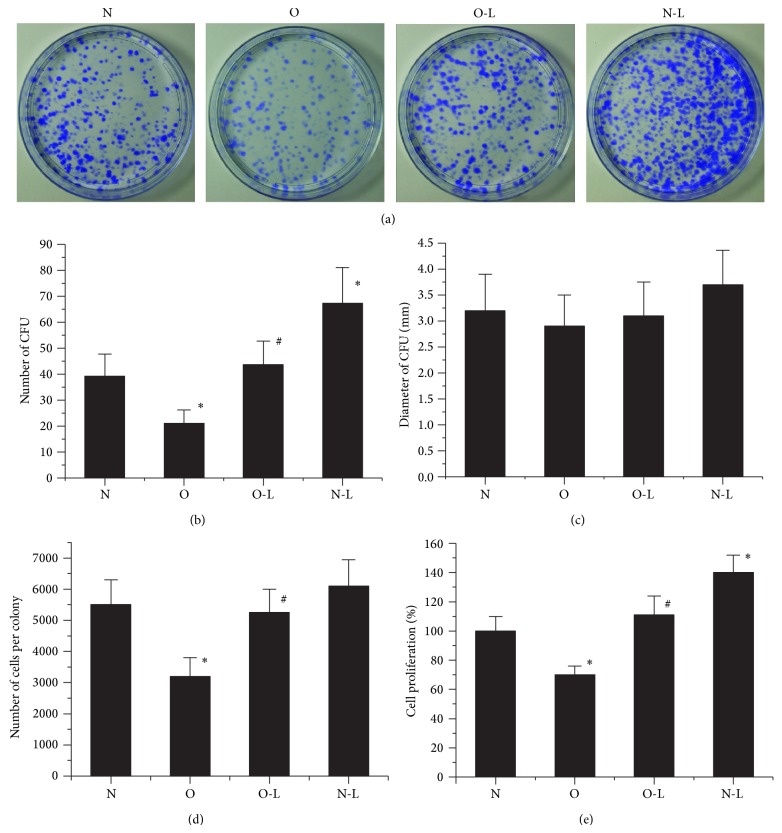
The colony-forming unit and proliferation of MSCs. (a) Representative photograph of colonies in CFU-A at day 7. (b) Number of CFU detected per 10^6^ of seeded cells in CFU-A. (c) Average diameter of CFU. (d) Average number of cells per colony in CFU-A. (e) Cell proliferation was measured by WST-1 assay. The optical density (OD) value of normal MSCs was used as 100% cell proliferation. The data were drawn from three independent experiments and the results were expressed as mean ± SD. ^*^ *P* < 0.05 versus N Group. ^#^ *P* < 0.05 versus O Group.

The number of CFU, mean diameter of the CFU, and number of cells per colony in cultures of each group are calculated (Figure 1). The number of CFU and number of cells per colony in cultures of O Group was significantly lower than those in the other three groups. Furthermore, there was no significant difference in the number of CFU and number of cells per colony between the N Group and the O-L Group. Compared with the N Group, the N-L Group showed increased number of CFU and number of cells per colony. There was no significant difference in the diameter of the CFU among the four groups. Proliferation of MSCs was assessed by WST-1 cell proliferation assay. The proliferation rate in the O-L Group was significantly higher than that in the O Group. Compared with N Group, the O Group proliferated significantly slower and the N-L Group proliferated significantly faster. Obtained results suggested that glucocorticoids suppressed proliferation of MSCs; however, low oxygen (2%) enhances the proliferation of MSCs from ONFH rabbits contributing to the increased growth rate of the total BMSC population.

### 3.2. Low Oxygen Culture Promoted Expression of Stemness Genes in MSCs

**Figure 2:**
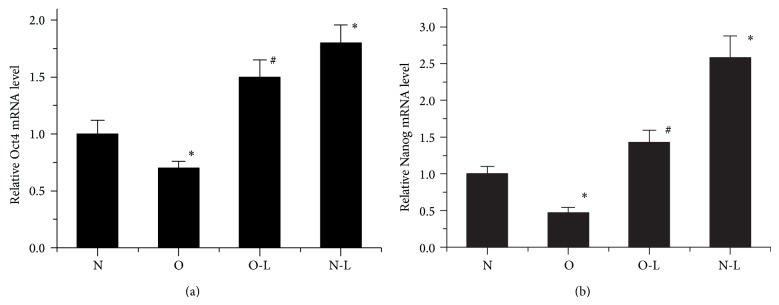
Effect of low oxygen on expression of stemness genes in MSCs. (a) Expression of Oct4 in MSCs 5 days after normoxia or low oxygen culture. (b) Expression of Nanog in MSCs of each group. The data were drawn from three independent experiments and the results were expressed as mean ± SD. ^*^ *P* < 0.05 versus N Group. ^#^ *P* < 0.05 versus O Group.

To explore the stemness of the osteonecrosis MSCs and the effect of low oxygen tension (2%) on stemness, we analyzed the expression of stemness gene, including Oct4 and Nanog, in MSCs of every group. RT-PCR analysis showed that the osteonecrosis MSCs have repressed stemness genes. Furthermore, low oxygen (2%) enhanced the stemness genes of both normal MSCs and osteonecrosis MSCs (Figure 2).

### 3.3. Inhibition of Adipogenesis of MSCs from Rabbits with ONFH after Low Oxygen (2%) Culture

**Figure 3:**
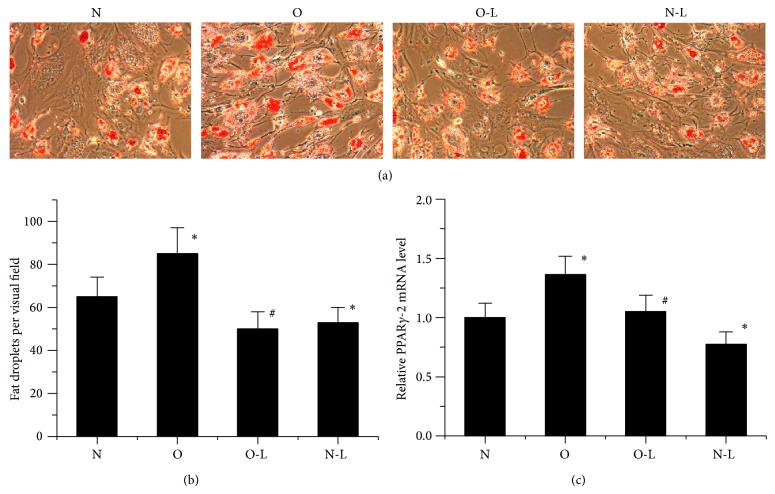
Effect of low oxygen on MSC adipogenic differentiation. (a) Oil Red O staining was performed to measure adipocyte formation 21 days after cells were induced in an adipogenic induction medium. (b) Fat droplets per visual field were determined. (c) Expression of PPAR*γ*-2 mRNA was determined by qRT-PCR at day 10. The data were drawn from three independent experiments and the results were expressed as mean ± SD. ^*^ *P* < 0.05 versus N Group. ^#^ *P* < 0.05 versus O Group.

To understand the effects of glucocorticoids and low oxygen (2%) on adipogenic differentiation, we induced bone marrow MSCs into adipocytes under induction medium conditions. We found, compared with normal MSCs, adipogenic differentiation of MSCs from osteonecrosis rabbits to be clearly promoted under normoxia (Figure 3(a)) indicated by Oil Red O staining. Furthermore, the expression of PPAR*γ*2 increased at mRNA level (Figure 3(c)). However, after adipogenesis induction in response to low oxygen (2%), the results from Oil Red O staining showed that the adipogenesis of MSCs from both ONFH rabbits and normal rabbits was downregulated at day 21 (Figure 3(a)). Consistently, there were less lipid droplets with the treatment of low oxygen (2%), and the number of fat droplets in the O-L Group was about 60 percent of the O Group (Figure 3(b)). RT-PCR further demonstrated that MSCs treated in AIM (adipogenic induction medium) under low oxygen (2%) showed a decreased pattern of expression of PPAR*γ*2 (Figure 3(c)). These data indicated that treatment with low oxygen (2%) inhibited adipogenic differentiation of MSCs from rabbits with ONFH.

### 3.4. Regulation of Osteogenic Differentiation by Low Oxygen (2%) Culture

**Figure 4:**
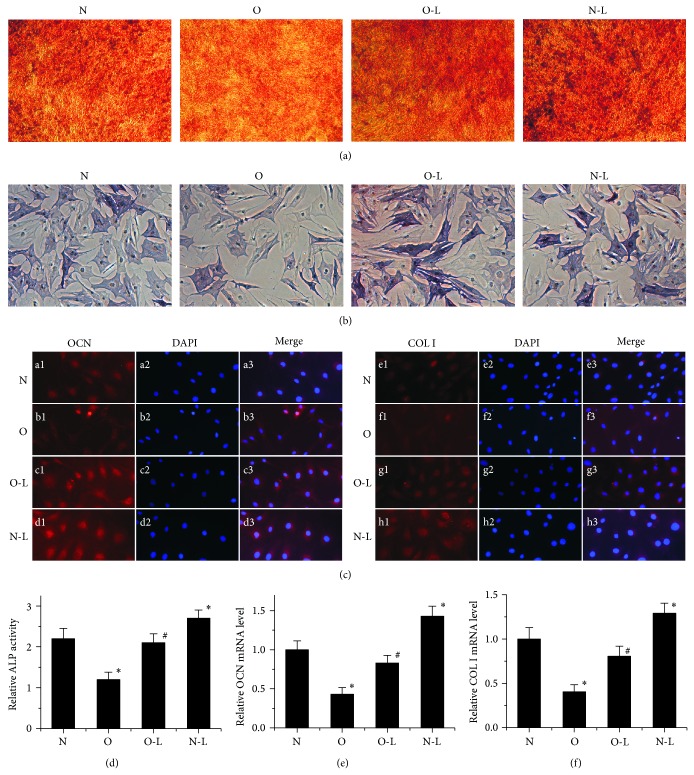
Effect of low oxygen (2%) on MSC osteogenic differentiation. (a) The mineralization nodes were monitored by Alizarin Red S staining 14 days after cells were induced in an osteogenic induction medium. (b) Alkaline phosphatase staining of MSCs after osteogenic differentiation. (c) Immunostaining for OCN and COL I (Texas-Red, a1-h1) with nuclear counterstained (DAPI-blue, a2-h2) 2 weeks after osteogenic differentiation (×400). (a3-h3) Merge. (d) ALP activities in BMSCs. (e) OCN mRNA expression was examined by RT-PCR at day 14. (f) COL I mRNA expression was examined by RT-PCR at day 14. The data were drawn from three independent experiments and the results were expressed as mean ± SD. ^*^ *P* < 0.05 versus N Group. ^#^ *P* < 0.05 versus O Group.

To determine the role of glucocorticoids and low oxygen (2%) on osteoblast differentiation of bone marrow derived mesenchymal stem cells, we examined the expression of osteoblast differentiation markers including ALP, OCN, and COL I 14 days after cultured in osteogenic induction medium under low oxygen (2%) and Alizarin Red S staining was performed. Alizarin Red S staining showed that the area of positive staining in N Group and O-L Group was greater compared with that in the O Group (Figure 4(a)). Compared with the N Group, the N-L Group showed increased area of Alizarin Red S-positive. Similar results were observed in ALP staining and ALP activities (Figures 4(b) and 4(d)). Moreover, we found that OCN and COL I were strongly expressed in MSCs in the O-L Group (Figures 4(c), 4(e), and 4(f)) compared with O Group. Compared with the N Group, the N-L Group exhibited enhanced expression of OCN and COL I at mRNA level (Figures 4(e) and 4(f)). These data indicated that glucocorticoids suppress osteogenic differentiation and treatment with low oxygen (2%) enhances osteogenic potential of bone marrow-derived mesenchymal stem cells from rabbits with glucocorticoids-induced osteonecrosis.

## 4. Discussion

In our study, it was found that the proliferation, stemness, and osteogenic differentiation potential of MSCs from osteonecrosis animals were impaired. Low oxygen (2%) could enhance the proliferation and maintenance of stemness and osteogenic differentiation potential of MSCs with osteonecrosis-related functional impairment.

Osteonecrosis of the femoral head (ONFH) is a debilitating orthopedic disease primarily affecting young and middle-aged patients. It is thought to be caused by death of bone cells due to ischemia and hypoxia. Current treatment options for early stage ONFH include electrical stimulation, core decompression, rotational osteotomy, and nonvascularized and vascularized bone grafting. The best approach for precollapse ONFH remains unanswered. Core decompression used to be the most widespread procedure to treat the ONFH; its efficacy still remains controversial. The vascularized fibular grafts have demonstrated the highest rates of success in treating early-stage ONFH, but there is still a great concern with its complications. This has prompted the investigation into a novel method for treatment of ONFH.

Mesenchymal stromal cells (MSCs) are pluripotent cells, capable of differentiating into a variety of cell types including osteoblasts, chondrocytes, adipocytes, and myoblasts. Therefore, cell therapy using MSCs represents a promising approach to promote wound healing and tissue regeneration, such as in repair of bone fractures. Recently, some groups of researchers transplanted MSCs into the necrotic area of the femoral head to aid the core compression in order to treat ONFH. Gangji et al. followed up 19 patients (24 hips) for five years and found BMCs transplantation could obviously release pain and improve the function of hips in patients with early stages of ONFH [14]. However, the successful repair of a large bone defect with cell transplantation therapy needs large numbers of functional MSCs cells.

It has been suggested recently that osteonecrosis of the femoral head might be a disease of MSCs, due to abnormal proliferation or differentiation of MSCs. In patients with osteonecrosis secondary to corticosteroid therapy, abnormalities have been demonstrated in the bone marrow of the iliac crest, with a decrease in the stem-cell pool [15]. Steroids have been shown to produce adipogenesis and to stimulate fat specific genes in cloned bone-marrow cells [16]. These findings suggest that impairments of BMSCs were responsible for osteonecrosis which might represent a cellular mechanism. As shown in the present study, proliferation rate, those osteogenic markers, stemness genes, and alkaline phosphatase activity and mineralization were significantly reduced in osteonecrosis BMSCs compared with normal BMSCs. On the contrary, those adipogenic markers and lipid droplets significantly increased in osteonecrosis BMSCs. Our data clearly revealed impaired proliferation, stemness, and osteogenic potential of osteonecrosis BMSCs, which helps to explain the well-documented correlation between the incidence of osteonecrosis and glucocorticoid administration. It also suggested that BMSCs augmentation could be a potential strategy in the treatment of osteonecrosis.

As mentioned before, BMSCs from patients with steroid-induced osteonecrosis showed impaired proliferation ability and osteogenic potential; it is necessary to find new ways to revise the impairments. Preconditioning of stem cells with growth factors [17, 18], heat shock [19], and antiaging compounds represents an effective strategy to enhance survival, proliferation, and differentiation of MSCs. Several studies have been carried out in order to analyze the effects of low oxygen on MSCs and beneficial effects of low oxygen culture on proliferation and* in vitro* and* in vivo* differentiation potentials of human MSCs have been suggested by several groups. Hung et al. demonstrated that human bone marrow-derived MSCs (hBM-MSCs) cultured under low oxygen (1%) had increased proliferation [12]. In our study, the WST-1 assay and colony formation unit assay were performed to investigate the effect of low oxygen (2%) on proliferation of MSCs from both ONFH rabbits and healthy rabbits. It was indicated that MSCs proliferation in groups under low oxygen (2%) increased obviously compared with those under normoxia which was in accordance with the findings of Ren et al. and Lennon et al. [20, 21]. Recent studies have indicated that low oxygen plays an important role in altering characteristics of various types of stem cells including embryonic stem cells [22], induced pluripotent stem cells [23], adipose tissue-derived stromal cells, and mesenchymal stem cells [24]. Yamamoto et al. found that low oxygen tension (2%) promotes the expression of Oct3/4 and Nanog (stem-cell marker) of adipose tissue-derived stromal cells [24]. In another study by Hung et al., it was demonstrated that human bone marrow-derived MSCs (hBM-MSCs) cultured under low oxygen (1%) had enhanced expression of stemness genes including OCT4, NANOG, SALL4, and KLF4 [12]. In our study, similar results were presented. We found that low oxygen (2%) promoted expression of stemness genes including OCT4 and Nanog in both osteonecrosis BMSCs and normal BMSCs, indicating that low oxygen tension (2%) promotes maintenance of stemness in MSCs.

Differentiation of MSCs under low oxygen favoured osteogenesis, while adipogenesis was inhibited [12, 25]. However, the investigations on the effect of low oxygen on MSC differentiation published by other authors show conflicting results [26, 27]. Taken together, the effects of low oxygen on MSCs differentiation varied and depended on oxygen tensions, cell source, and species. Given this background, we investigated the effect of low oxygen (2%) on differentiation potential of MSCs from both ONFH rabbits and healthy rabbits towards adipogenic and osteogenic lineages. With regard to our defined experimental conditions we found that adipogenesis was suppressed by low oxygen (2%), whereas osteogenesis was enhanced. These data were supported by Lennon et al. and Boyette et al. [21, 28].

In conclusion, we have shown that the proliferation, stemness, and osteogenic differentiation potential of MSCs from osteonecrosis rabbits were depressed, and the adipogenic differentiation potential was promoted. Moreover, low oxygen (2%) culture could enhance the proliferation, expression of stemness genes, and osteogenic differentiation potential of MSCs from both ONFH rabbits and healthy rabbits and inhibit the adipogenic differentiation. Low oxygen (2%) culture may therefore serve as a useful strategy to augment BMMSC function affected by glucocorticoids and holds significance for future strategies to treat femoral head osteonecrosis.
